# Development of a Clinical Prediction Model for 1-Year Mortality in Patients With Advanced Cancer

**DOI:** 10.1001/jamanetworkopen.2022.44350

**Published:** 2022-11-30

**Authors:** Catherine Owusuaa, Annemieke van der Padt-Pruijsten, Jan C. Drooger, Joan B. Heijns, Anne-Marie Dietvorst, Ellen C. J. Janssens-van Vliet, Daan Nieboer, Joachim G. J. V. Aerts, Agnes van der Heide, Carin C. D. van der Rijt

**Affiliations:** 1 Department of Medical Oncology, Erasmus MC Cancer Institute, Rotterdam, the Netherlands; 2 Department of Internal Medicine, Maasstad Hospital, Rotterdam, the Netherlands; 3 Department of Internal Medicine, Ikazia Hospital, Rotterdam, the Netherlands; 4 Department of Internal Medicine, Amphia, Breda, the Netherlands; 5 Department of Internal Medicine, Van Weel Bethesda Hospital, Dirksland, the Netherlands; 6 Department of Internal Medicine, Admiraal de Ruyter Hospital, Goes, the Netherlands; 7 Department of Public Health, Erasmus MC, Erasmus University Medical Center, Rotterdam, the Netherlands; 8 Department of Pulmonary Diseases, Erasmus MC, Erasmus University Medical Center, Rotterdam, the Netherlands

## Abstract

**Importance:**

To optimize palliative care in patients with cancer who are in their last year of life, timely and accurate prognostication is needed. However, available instruments for prognostication, such as the surprise question (“Would I be surprised if this patient died in the next year?”) and various prediction models using clinical variables, are not well validated or lack discriminative ability.

**Objective:**

To develop and validate a prediction model to calculate the 1-year risk of death among patients with advanced cancer.

**Design, Setting, and Participants:**

This multicenter prospective prognostic study was performed in the general oncology inpatient and outpatient clinics of 6 hospitals in the Netherlands. A total of 867 patients were enrolled between June 2 and November 22, 2017, and followed up for 1 year. The primary analyses were performed from October 9 to 25, 2019, with the most recent analyses performed from June 19 to 22, 2022. Cox proportional hazards regression analysis was used to develop a prediction model including 3 categories of candidate predictors: clinician responses to the surprise question, patient clinical characteristics, and patient laboratory values. Data on race and ethnicity were not collected because most patients were expected to be of White race and Dutch ethnicity, and race and ethnicity were not considered as prognostic factors. The models’ discriminative ability was assessed using internal-external validation by study hospital and measured using the C statistic. Patients 18 years and older with locally advanced or metastatic cancer were eligible. Patients with hematologic cancer were excluded.

**Main Outcomes and Measures:**

The risk of death by 1 year.

**Results:**

Among 867 patients, the median age was 66 years (IQR, 56-72 years), and 411 individuals (47.4%) were male. The 1-year mortality rate was 41.6% (361 patients). Three prediction models with increasing complexity were developed: (1) a simple model including the surprise question, (2) a clinical model including the surprise question and clinical characteristics (age, cancer type prognosis, visceral metastases, brain metastases, Eastern Cooperative Oncology Group performance status, weight loss, pain, and dyspnea), and (3) an extended model including the surprise question, clinical characteristics, and laboratory values (hemoglobin, C-reactive protein, and serum albumin). The pooled C statistic was 0.69 (95% CI, 0.67-0.71) for the simple model, 0.76 (95% CI, 0.73-0.78) for the clinical model, and 0.78 (95% CI, 0.76-0.80) for the extended model. A nomogram and web-based calculator were developed to support clinicians in adequately caring for patients with advanced cancer.

**Conclusions and Relevance:**

In this study, a prediction model including the surprise question, clinical characteristics, and laboratory values had better discriminative ability in predicting death among patients with advanced cancer than models including the surprise question, clinical characteristics, or laboratory values alone. The nomogram and web-based calculator developed for this study can be used by clinicians to identify patients who may benefit from palliative care and advance care planning. Further exploration of the feasibility and external validity of the model is needed.

## Key Points

**Question:**

Can a predictive model be developed and validated to calculate the 1-year risk of death among patients with advanced cancer by combining clinician responses to the surprise question (“Would I be surprised if this patient died in the next year?”) with patients’ clinical characteristics and laboratory values?

**Findings:**

In this prognostic study that included 867 patients with advanced cancer, the developed model combining the surprise question, clinical characteristics, and laboratory values had better discriminative ability in predicting 1-year risk of death than the surprise question, clinical characteristics, or laboratory values alone. A nomogram was developed to aid clinicians in identifying those at risk of dying within 1 year.

**Meaning:**

These results suggest that the prediction model and nomogram developed for this study can be used by clinicians to identify patients who may benefit from palliative care and advance care planning.

## Teaser

This prognostic study of patients with cancer in the Netherlands describes the development and internal-external validation of a prognostic model to calculate the 1-year risk of death among patients with advanced cancer.

## Introduction

Palliative care aims to optimize the quality of life among both patients who are in the last phase of life and their relatives.^1,2^ High-quality and patient-centered palliative care is supported by timely advance care planning.^3,4^ Palliative care and advance care planning can be facilitated by adequate prognostication (ie, making predictions about a patient’s remaining life expectancy).^5^ Prognostication may be based on clinicians’ subjective predictions, objective predictors or prediction models, or both.

The surprise question (“Would I be surprised if this patient died in the next year?”) is a well-known tool to support clinician prediction of the survival of patients with advanced illness. It is a generic non–disease-specific tool that is recommended to identify patients with palliative care needs.^6^ The surprise question alone has been studied as a predictor of death within 1 year among patients with cancer and found to have a sensitivity of 75% and specificity of 90%.^7^ Those findings suggest that the surprise question is suitable for identifying patients with cancer who will live beyond 1 year but less suitable for identifying those who are going to die within 1 year. Mudge et al^8^ attempted to improve the prognostic performance of the surprise question for 1-year mortality among hospital inpatients by combining the surprise question with indicators of functional deterioration.^9^ These indicators included general deterioration (eg, declining functional performance status, weight loss, or repeated unplanned hospital admissions) and clinical indicators for specific advanced diseases (eg, functional ability deteriorating due to progressive cancer or heart failure or extensive untreatable coronary artery disease, with breathlessness or chest pain at rest or on minimal effort). The surprise question combined with general and disease-specific indicators had higher accuracy in predicting death within 1 year than the surprise question alone (81.3% vs 62.0%).^8^

Cancer-specific prediction models, such as the Palliative Prognostic Score and the Palliative Prognostic Index, have been widely studied and validated for predicting whether patients are in the last months, weeks, or days of life.^10^ However, few studies have investigated predictors or prediction models for the last year of life. A review by Owusuaa et al^11^ summarized predictors of death within 3 months to 2 years; these predictors included age, sex, Eastern Cooperative Oncology Group (ECOG) performance status, brain metastases, visceral metastases, and cutaneous or subcutaneous metastases. Prediction models consisting of 1 or more predictors (eg, the Oncological-Multidimensional Prognostic Index) identified in this review did not include any form of clinician prediction of survival. Furthermore, those models had moderate discrimination abilities (C statistic or area under the curve of 0.60-0.70) or were not well (ie, externally) validated.^11^

It is well established that prognostication is most accurate when clinician predictions of survival are combined with clinical predictors.^12^ However, little is known about that combination for the prediction of death within 1 year in patients with cancer. Therefore, we aimed to develop and validate a model to calculate the 1-year risk of death for patients with advanced cancer.

## Methods

### Patients and Procedures

The protocol for this prognostic study was reviewed and approved by the medical ethical research committee of Erasmus MC, Erasmus University Medical Center, Rotterdam. The study protocol was also approved by the other study hospitals. All eligible patients were informed about the study in writing, and written or oral informed consent (depending on the procedure of the study hospital) was obtained from all patients. The collected data were analyzed anonymously. This study followed the Transparent Reporting of a Multivariable Prediction Model for Individual Prognosis or Diagnosis (TRIPOD) reporting guideline for prognostic studies.^13^

Patients eligible for inclusion were 18 years or older, had locally advanced or metastatic cancer, and were receiving treatment with palliative intent, with or without anticancer treatment. A total of 867 patients (847 from outpatient clinics) were prospectively and consecutively enrolled from both the general oncology inpatient clinics and the outpatient clinics of 6 hospitals in the Netherlands (Erasmus MC, Ikazia Hospital Rotterdam, Maasstad Hospital Rotterdam, Amphia, Van Weel Bethesda Hospital, and Admiraal de Ruyter Hospital) from June 2 to November 22, 2017 (eBox 1 in Supplement 1). Patients with hematologic cancer were excluded. Medical specialists, residents, and nurse practitioners from the study hospitals enrolled eligible patients consecutively based on the 3 inclusion criteria and 1 exclusion criterion, which were outlined on a poster in every consultation room and clinic. The primary analyses were performed from October 9 to 25, 2019, with the most recent analyses performed from June 19 to 22, 2022.

A total of 17 candidate predictors of death were selected based on the findings of a systematic review and meta-analysis.^11^ These predictors were categorized as follows: (1) clinician responses to the surprise question (“Would I be surprised if this patient died in the next year?”^7^); (2) patient clinical characteristics, including age, sex, comorbidity, cancer type, metastases (visceral [including liver, pancreas, peritoneal, or pleural and excluding lung], brain, and cutaneous or subcutaneous), ECOG performance status, food intake, weight loss, pain, dyspnea, and fatigue; and (3) patient laboratory values, including hemoglobin, C-reactive protein (CRP), and serum albumin (eBox 2 in Supplement 1). Data on race and ethnicity were not collected because most patients were expected to be of White race and Dutch ethnicity, and race and ethnicity were not considered as prognostic factors. Before this study began, the clinical feasibility of collecting information on these predictors was evaluated in a focus group comprising oncologists and other clinicians.

Variables were gathered on the day of inclusion via a questionnaire, which was completed by the medical specialist, resident, or nurse practitioner who was treating the patient. The questionnaire included the surprise question and items about the patient’s current performance status, which was assessed according to the ECOG classification system (range, 0-4, with 0 indicating no performance restrictions and 4 indicating totally confined to bed or chair)^14^; the patient’s current food intake (normal, mildly reduced, or severely reduced), which was evaluated by asking the patient; the patient’s average pain during the previous week, which was assessed using an 11-point numerical rating scale (range, 0-10, with 0 indicating no pain and 10 indicating the worst pain possible)^15^; the patient’s level of dyspnea (range, 0-4, with 0 indicating no dyspnea and 4 indicating life-threatening dyspnea) and level of fatigue (range, 0-3, with 0 indicating no fatigue and 3 indicating fatigue that limits self-care activities of daily living), which were assessed according to the Common Terminology Criteria for Adverse Events, version 4.0^16^; and the patient’s weight loss, which was assessed by asking the patient about total weight loss in the previous 6 months. Research assistants obtained information on other variables from patients’ medical records. Comorbidity was assessed using the Charlson Comorbidity Index.^17^

For the laboratory parameters, the most recent test results from the month before study inclusion were collected. Types of cancer were classified based on literature^18,19^ as those having (1) a good prognosis (mean survival of 40 months) for breast, prostate, or thyroid cancer or (2) an intermediate or poor prognosis (mean survival of 10-24 months) for all other cancer types. The sample size was estimated at 430 patients, which was based on the expected mortality rate (40%) and the number of expected deaths (170) in relation to the total number of predictors (17).^11,20^ All patients were followed up for a maximum of 1 year, and information about their vital status (ie, alive or dead) was obtained from medical records. When it was unclear whether the patient was still alive, the patient’s general practitioner was contacted by telephone. For patients who died during follow-up, the date of death was recorded.

### Statistical Analysis

The primary outcome was the probability of death by 1 year. The prognostic performance of the surprise question in predicting death within 1 year was assessed. Possible nonlinear associations between the risk of death and continuous predictors were investigated using restricted cubic splines. If there was evidence of a nonlinear association, a suitable transformation was chosen to approximate the spline. Cox proportional hazards regression analysis was used to develop a prediction model by applying backward selection using a liberal *P* value (*P* < .20). We assumed that missing values were missing at random; multiple imputation was used to impute missing values 10 times. The results from analyses of these imputed data sets were pooled using Rubin rules.^21^ We performed sensitivity analyses to examine the possible impact of the violation of missing at random.

The prediction model was validated through internal-external validation to evaluate heterogeneity in model performance across the study hospitals.^22^ In this validation, the prediction model was refitted with data from all study hospitals except 1, and the resulting model was validated with the data from the hospital not included at model development. This procedure was repeated until each hospital was used once for validation. We used the Harrell C statistic to evaluate the ability of the prediction model to discriminate between patients who died vs patients who lived longer during the follow-up period. The C statistic ranges from 0.5 to 1.0, with 0.5 indicating that a model yields prognostic results equivalent to a coin toss and 1.0 indicating that a model has perfect prognostic discrimination. In addition, we assessed the calibration of the model during internal-external validation using calibration plots.

We also simplified the prediction model into a nomogram, and we created a web-based calculator to calculate the probability of death within 1 year. All statistical analyses were performed using R statistical software, version 3.6.0 (R Foundation for Statistical Computing). Missing values were imputed using the mice package for R software. The web-based calculator was developed using the Shiny package for R software. The threshold for statistical significance was 2-sided *P* = .20.

## Results

**Figure 1.**
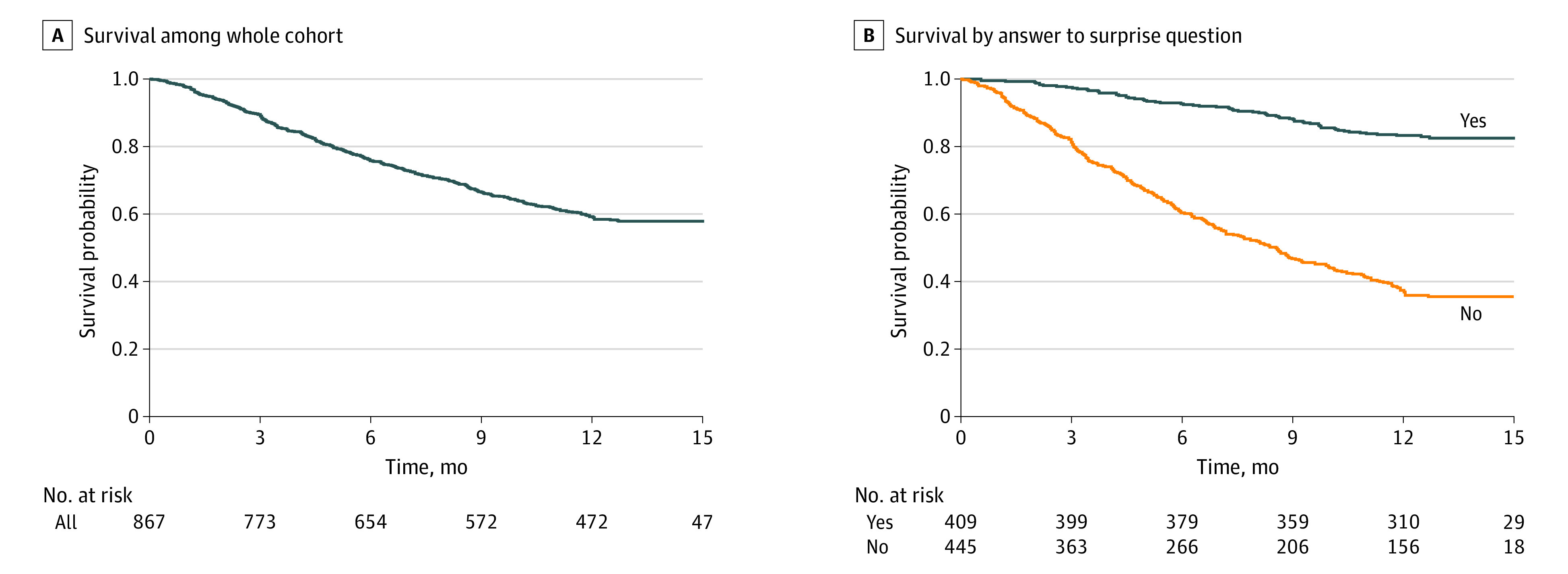
Kaplan-Meier Survival Curve Survival among 867 patients with advanced cancer.

Among 867 patients, the median age was 66 years (IQR, 56-72 years); 456 patients (52.6%) were female, and 411 patients (47.4%) were male. Most patients (476 individuals [54.9%]) were enrolled at 1 university-affiliated tertiary hospital (Erasmus MC). The most common cancer types were breast (191 patients [22.0%]), lung (173 patients [20.0%]), and gastrointestinal (132 patients [15.2%]) (Table 1; eTable 1 in Supplement 1). Of all cancer types, 595 (68.6%) had an intermediate or poor prognosis according to data from the literature.^18,19^ The 1-year mortality rate within the whole cohort was 41.6% (361 patients) (Figure 1). Although there were no missing data on vital status, we had to contact the patient’s general practitioner to ascertain the outcomes of 77 patients (8.9%). The 1-year survival probability was 82% among patients for whom clinicians answered *yes* to the surprise question and 37% among patients for whom clinicians answered *no* to the surprise question.

**Table 1.** Patient Characteristics

Characteristic	Patients, No./total No. (%) (N = 867)
Study hospital	
Erasmus MC	476/867 (54.9)
Ikazia Hospital Rotterdam	133/867 (15.3)
Maasstad Hospital Rotterdam	77/867 (8.9)
Amphia	104/867 (12.0)
Van Weel Bethesda Hospital	29/867 (3.3)
Admiraal de Ruyter Hospital	48/867 (5.5)
Age, median (IQR), y	66 (56-72)
Outpatient	847/867 (97.7)
Sex	
Female	456/867 (52.6)
Male	411/867 (47.4)
Clinician response of *no* to surprise question^a^	445/854 (52.1)
Respondents to surprise question	
Medical specialists	767/867 (88.5)
Nurse practitioners	55/867 (6.3)
Residents	45/867 (5.2)
ECOG performance status	
0	264/864 (30.6)
1	432/864 (50.0)
≥2	168/864 (19.4)
Cancer type	
Breast	191/867 (22.0)
Lung	173/867 (20.0)
Gastrointestinal	132/867 (15.2)
Prostate	76/867 (8.8)
Melanoma	60/867 (6.9)
Gynecological	53/867 (6.1)
Pancreas	40/867 (4.6)
Thyroid	5/867 (0.6)
All other types	137/867 (15.8)
Cancer type prognosis	
Good	272/867 (31.4)
Intermediate or poor	595/867 (68.6)
Visceral metastases	347/867 (40.0)
Brain metastases	66/867 (7.6)
Cutaneous or subcutaneous metastases	40/867 (4.6)
Food intake	
Normal	640/854 (74.9)
Lightly reduced	159/854 (18.6)
Strongly reduced	55/854 (6.4)
Weight loss, median (IQR), kg^b^	0 (0-2)
Pain score, median (IQR)^c^^,^^d^	0 (0-3)
Dyspnea level^e^	
0	570/867 (65.7)
1	225/867 (26.0)
≥2	72/867 (8.3)
Fatigue level^e^	
0	256/866 (29.6)
1	484/866 (55.9)
≥2	126/866 (14.5)
Charlson Comorbidity Index score	
0	570/867 (65.7)
1	208/867 (24.0)
≥2	89/867 (10.3)
Hemoglobin, median (IQR), g/dL^f^	12.4 (11.1-13.7)
C-reactive protein, median (IQR), mg/dL^g^	0.62 (0.25-2.60)
Serum albumin, median (IQR), g/dL^h^	4.1 (3.8-4.3)
Dead at 1 y	361/867 (41.6)

Abbreviation: ECOG, Eastern Cooperative Oncology Group.

SI conversion factors: To convert hemoglobin from grams per deciliter to millimoles per liter, divide by 1.6113; to convert C-reactive protein from milligrams per deciliter to milligrams per liter, multiply by 10; to convert serum albumin from grams per deciliter to grams per liter, multiply by 10.

^a^ The surprise question was, “Would I be surprised if this patient died in the next year?”

^b^ Data were missing for 33 patients (3.8%).

^c^ Pain was assessed using an 11-point numerical rating scale (score range, 0-10, with 0 indicating no pain and 10 indicating the worst pain possible).

^d^ Data were missing for 8 patients (0.9%).

^e^ Dyspnea and fatigue levels were assessed using the Common Terminology Criteria for Adverse Events, version 4.0. For dyspnea, the range was 0 to 4, with 0 indicating no dyspnea and 4 indicating life-threatening dyspnea; for fatigue, the range was 0 to 3, with 0 indicating no fatigue and 3 indicating fatigue that limits self-care activities of daily living.

^f^ Data were missing for 77 patients (8.9%).

^g^ Data were missing for 365 patients (42.1%).

^h^ Data were missing for 168 patients (19.4%).

The surprise question was answered mainly by attending medical specialists (767 patients [88.5%]), followed by nurse practitioners (55 patients [6.3%]) and residents (45 patients [5.2%]). There were no significant differences between these groups in the prognostic accuracy of the prediction of 1-year death. The surprise question had a hazard ratio (HR) of 5.42 (95% CI, 5.27-7.16) when answered by specialists, 3.86 (95% CI, 3.50-10.24) when answered by nurse practitioners, and 7.32 (95% CI, 6.48-24.66) when answered by residents (*P =* .61). Overall, the surprise question had a sensitivity of 80% (95% CI, 75%-84%), specificity of 68% (95% CI, 90%-95%), positive predictive value (PPV) of 64% (95% CI, 60%-68%), and negative predictive value (NPV) of 82% (95% CI, 79%-86%). In the univariable regression model, all variables (except for the presence of cutaneous or subcutaneous metastases) were associated with death within 1 year, with the highest risk observed for a clinician answer of *no* to the surprise question (HR, 5.49; 95% CI, 4.22-7.13), an ECOG performance status of 2 or higher (HR, 4.67; 95% CI, 3.47-6.27), and a fatigue grade of 2 or higher (HR, 4.29; 95% CI, 3.12-5.91)(Table 2).

**Table 2.** Univariate Analysis of Surprise Question Responses, Clinical Characteristics, and Laboratory Values

Variable	HR (95% CI)	*P* value
Clinician response to surprise question		
Yes	1 [Reference]	NA
No	5.49 (4.22-7.13)	<.001
Age (per 10-y increase)	1.07 (0.98-1.17)	.14
Sex		
Female	1 [Reference]	NA
Male	1.35 (1.10-1.66)	.004
ECOG performance status		
0	1 [Reference]	NA
1	1.64 (1.24-2.17)	<.001
≥2	4.67 (3.47-6.27)	<.001
Cancer type prognosis		
Good	1 [Reference]	NA
Intermediate or poor	1.99 (1.55-2.56)	<.001
Visceral metastases	1.42 (1.16-1.75)	.001
Brain metastases	1.71 (1.22-2.40)	.004
Cutaneous or subcutaneous metastases	0.87 (0.52-1.46)	.58
Food intake		
Normal	1 [Reference]	NA
Reduced	2.54 (2.05-3.14)	<.001
Weight loss		
No	1 [Reference]	NA
Yes	2.18 (1.76-2.70)	<.001
Weight loss (per kg)	1.07 (1.05-1.09)	<.001
Pain score (per U increase)^a^	1.11 (1.07-1.15)	<.001
Dyspnea level^b^		
0	1 [Reference]	NA
1	1.64 (1.30-2.07)	<.001
≥2	2.78 (2.03-3.80)	<.001
Fatigue level^b^		
0	1 [Reference]	NA
1	1.86 (1.41-2.45)	<.001
≥2	4.29 (3.12-5.91)	<.001
Charlson Comorbidity Index score		
0	1 [Reference]	NA
1	1.48 (1.16-1.87)	<.001
≥2	1.66 (1.21-2.27)	<.001
Hemoglobin (per U increase)	0.58 (0.51-0.66)	<.001
C-reactive protein (per doubling)	1.33 (1.25-1.41)	<.001
Serum albumin (per U increase)	0.92 (0.91-0.94)	<.001

Abbreviations: ECOG, Eastern Cooperative Oncology Group; HR, hazard ratio; NA, not applicable.

^a^ Pain was assessed using an 11-point numerical rating scale (score range, 0-10, with 0 indicating no pain and 10 indicating the worst pain possible).

^b^ Dyspnea and fatigue levels were assessed using the Common Terminology Criteria for Adverse Events, version 4.0. For dyspnea, the range was 0 to 4, with 0 indicating no dyspnea and 4 indicating life-threatening dyspnea; for fatigue, the range was 0 to 3, with 0 indicating no fatigue and 3 indicating fatigue that limits self-care activities of daily living.

In the multivariable analyses, we developed a prediction model for death within 1 year by increasing complexity, starting with the surprise question, which performed best in the univariable model. Three versions of the prediction model were developed: (1) a simple model including the surprise question only, (2) a clinical model including the surprise question and clinical characteristics (age, cancer type prognosis, visceral metastases, brain metastases, ECOG performance status, weight loss, pain, and dyspnea), and (3) an extended model including the surprise question, clinical characteristics, and laboratory values (hemoglobin, CRP, and serum albumin) (Table 3). The pooled C statistic was 0.69 (95% CI, 0.67-0.71) for the simple model, 0.76 (95% CI, 0.73-0.78) for the clinical model, and 0.78 (95% CI, 0.76-0.80) for the extended model (eTable 2 and eTable 3 in Supplement 1). At a uniform predefined 40% threshold for the risk of death, the clinical model had a sensitivity of 80%, specificity of 69%, PPV of 65%, and NPV of 83%. At this threshold, the extended model had a sensitivity of 76%, specificity of 72%, PPV of 66%, and NPV of 81%. The clinical and extended models had good calibration (eFigures 1 and 2 in Supplement 1).

**Table 3.** Cox Proportional Hazards Regression Analysis of Simple, Clinical, and Extended Prediction Models

Variable	HR (95% CI)		
	Simple model^a^	Clinical model^b^	Extended model^c^
Clinician response to surprise question			
Yes	1 [Reference]	1 [Reference]	1 [Reference]
No	5.49 (4.22-7.13)	3.80 (2.86-5.05)	3.46 (2.58-4.66)
Clinical characteristics			
Age (per 10-y increase)	NA	1.08 (0.98-1.19)	1.08 (0.98-1.19)
Cancer type prognosis			
Good	NA	1 [Reference]	1 [Reference]
Intermediate or poor	NA	1.45 (1.11-1.89)	1.40 (1.06-1.85)
Visceral metastases	NA	1.37 (1.11-1.70)	1.29 (1.04-1.60)
Brain metastases	NA	1.39 (0.98-1.96)	1.51 (1.05-2.17)
ECOG performance status			
0	NA	1 [Reference]	1 [Reference]
1	NA	1.04 (0.77-1.40)	0.96 (0.71-1.31)
≥2	NA	1.79 (1.27-2.54)	1.38 (0.96-2.00)
Weight loss			
Yes	NA	1 [Reference]	1 [Reference]
No	NA	1.26 (1.00-1.59)	1.11 (0.87-1.43)
Pain score (per U increase)^d^	NA	1.06 (1.01-1.10)	1.04 (0.99-1.09)
Dyspnea level^e^			
0	NA	1 [Reference]	1 [Reference]
1	NA	1.24 (0.98-1.59)	1.21 (0.95-1.54)
≥2	NA	1.51 (1.07-2.11)	1.29 (0.91-1.84)
Laboratory values			
Hemoglobin (per U increase)	NA	NA	0.88 (0.76-1.02)
C-reactive protein (per doubling)	NA	NA	1.12 (1.03-1.21)
Serum albumin (per U increase)	NA	NA	0.98 (0.95-1.01)

Abbreviations: ECOG, Eastern Cooperative Oncology Group; HR, hazard ratio; NA, not applicable.

^a^ The simple model included the surprise question (“Would I be surprised if this patient died in the next year?”).

^b^ The clinical model included the surprise question and clinical characteristics (age, cancer type prognosis, visceral metastases, brain metastases, ECOG performance status, weight loss, pain, and dyspnea).

^c^ The extended model included the surprise question, clinical characteristics, and laboratory values (hemoglobin, C-reactive protein, and serum albumin).

^d^ Pain was assessed using an 11-point numerical rating scale (score range, 0-10, with 0 indicating no pain and 10 indicating the worst pain possible).

^e^ Dyspnea level was assessed using the Common Terminology Criteria for Adverse Events, version 4.0. The range was 0 to 4, with 0 indicating no dyspnea and 4 indicating life-threatening dyspnea.

Additional analyses yielded a C statistic of 0.70 (95% CI, 0.68-0.73) for clinical characteristics alone, 0.71 (95% CI, 0.68-0.74) for laboratory values alone, and 0.77 (95% CI, 0.74-0.79) for the surprise question combined with laboratory values (eTable 3 in Supplement 1). Additional sensitivity analyses for CRP with a high percentage of missing values (42.1%) revealed no differences between imputed and complete-case analyses (eFigure 3 in Supplement 1).

**Figure 2.**
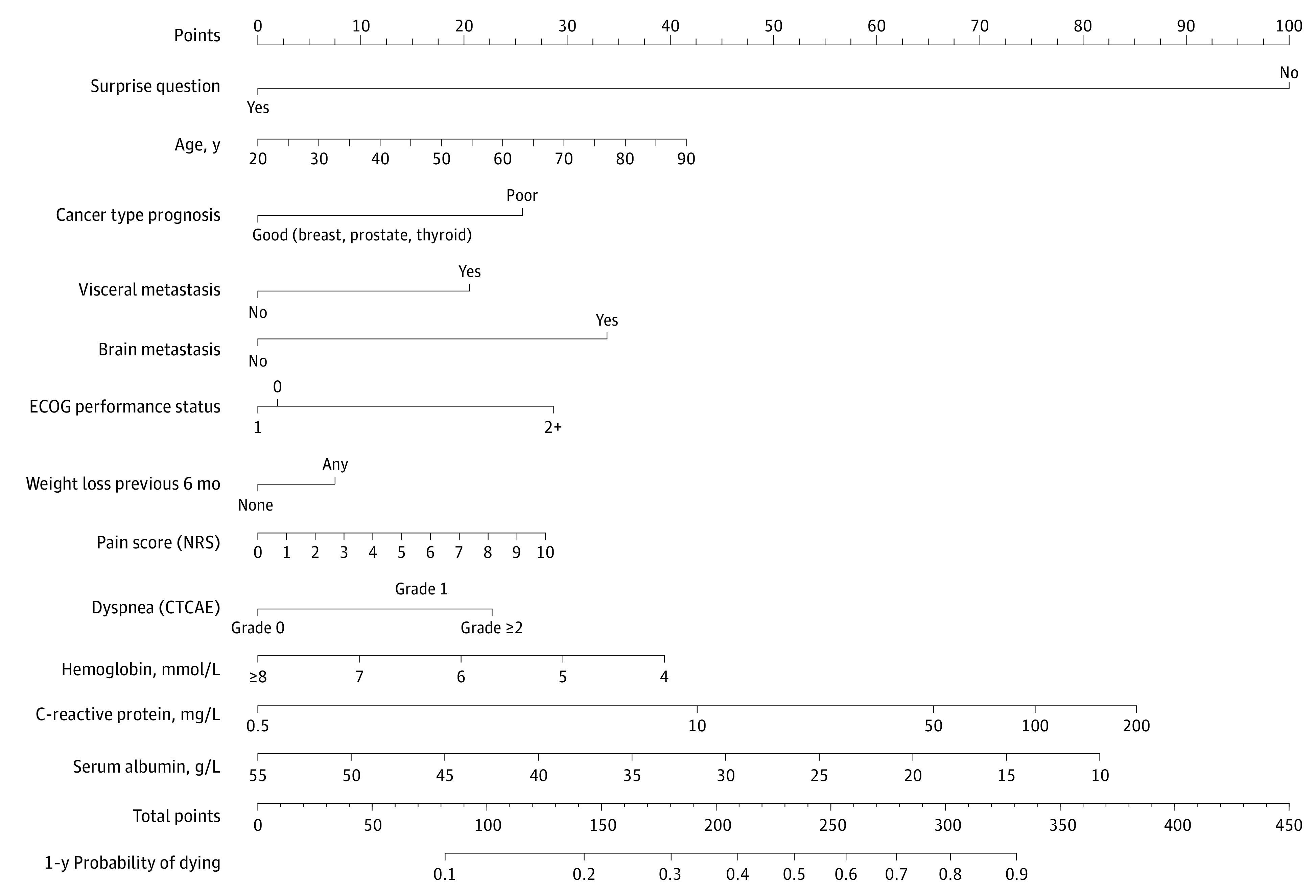
Nomogram of the Extended Model The surprise question was, “Would I be surprised if this patient died in the next year?” Instructions for use of the nomogram: (1) locate the answer to the surprise question, (2) draw a straight line upward to the point axis, (3) repeat this procedure for the other 11 predictors (age, cancer type prognosis, visceral metastases, brain metastasis, ECOG performance status, weight loss, pain, dyspnea, hemoglobin, C-reactive protein, and serum albumin), (4) sum the points for all predictors on the total points axis, and (5) draw a line straight down to the 1-year probability of dying axis to find the patient’s risk of dying within 1 year. Conventional unit conversion factors: To convert hemoglobin from millimoles per liter to grams per deciliter, multiply by 1.6113; to convert C-reactive protein from milligrams per liter to milligrams per deciliter, divide by 10; to convert serum albumin from grams per liter to grams per deciliter, divide by 10. CTCAE indicates Common Terminology Criteria for Adverse Events; ECOG, Eastern Cooperative Oncology Group; and NRS, numerical rating scale.

A nomogram, which calculated the 1-year risk of death based on individual variables, was developed for the simple model (eFigure 4 in Supplement 1), clinical model (eFigure 5 in Supplement 1), and extended model (Figure 2). A web-based calculator based on the models was also created.^23^ A sample calculation of 1-year risk of death based on 1 patient is shown in eBox 3 in Supplement 1.

## Discussion

This multicenter prospective prognostic study aimed to develop and perform internal-external validation on a prediction model for death by 1 year in patients with advanced cancer. We found that the extended model (including the surprise question, clinical characteristics, and laboratory values) had better discrimination ability than the simple model (including the surprise question only) or the clinical model (including the surprise question and clinical characteristics). However, the discriminative abilities of the clinical and extended models were relatively similar (C statistics of 0.76 and 0.78, respectively). The extended model developed in our study also had better discrimination than most other models in the literature.^11,24^ In addition, the clinical model had better discriminative ability than the simple model. Based on these results, our study confirmed previous findings that clinical and laboratory factors add to clinician prediction of survival using the surprise question.^12^

The development of an easy-to-use nomogram and web-based calculator for the clinical and extended models was novel and allowed for the calculation of 1-year risk of death in individual patients in clinical practice. Clinicians can choose to use the simple, clinical, or extended nomogram based on available patient information. However, the use of the clinical or extended model requires more variables than the 1-sentence surprise question. Because patients’ clinical characteristics may be easier to obtain than laboratory values, which require additional blood tests, clinician use of the extended model may be limited. Although the extended model best predicted the 1-year risk of death, the clinical model may be a good alternative due to the models’ similarities in discrimination (C statistics of 0.76 for the clinical model and 0.78 for the extended model).

The nomogram could be made visible in the patient’s electronic medical records and serve as a reminder for clinicians (both physicians and nurses) to be aware of patients who are at risk of dying within 1 year or could be implemented as part of a digital advance care planning program. The nomogram can support clinicians in initiating conversations with patients who may be in the last period of their lives and can thereby support advance care planning. The nomogram could also be an aid in making decisions about anticancer treatments in the last year of life. The interpretation of the calculated risk of death will need further research. We have yet to establish the threshold for risk of death that clinicians (should) feel comfortable using to communicate to patients that they may be in the last period of their lives, which could help to better tailor treatment decisions to quality of life.

Previous research^25^ has found that a machine learning algorithm, which used 559 features as inputs and was integrated into the electronic medical file, could accurately predict death within 180 days in patients with cancer. The study reported an area under the curve of 0.89,^25^ which outperformed our extended model. Of note, clinicians are already aware of the variables included in our model, whereas this awareness may not be the case with the variables included in a machine learning algorithm. Thus, it will be important to further assess support among health care professionals for the various types of prognostic models (a simple model, our model including multiple components, or a machine learning algorithm) in routine clinical practice.

In our study, the surprise question had higher sensitivity (80%) and higher PPV (64%) than previously reported (77% and 41%, respectively).^7,26^ The surprise question in our study was answered by clinicians in a hospital setting and applied to patients with advanced cancer, whereas other studies have often involved clinicians in the primary care setting and patients with all cancer stages. The surprise question may be easier to answer for patients with advanced cancer who typically have a worse prognosis than patients with other cancer stages. In addition, in contrast to previous findings,^27^ there were no significant differences between medical specialists and nurses with regard to the prognostic accuracy of the surprise question in predicting death within 1 year. The nurse practitioners in our study had more responsibility to assess and make decisions about the care of patients than nurses in a previous study,^27^ who seemed to be mainly involved in administering chemotherapy. Therefore, nurse practitioners may have expertise in answering the surprise question that is similar to that of medical specialists.

### Limitations

This study has several limitations. First, clinicians in our study enrolled eligible patients by completing a questionnaire. Although the 3 inclusion criteria and 1 exclusion criterion were clear, some bias in clinicians’ selection of patients cannot be ruled out. Second, the responses to the surprise question and information about other patient variables were collected within 1 questionnaire, which might have influenced clinicians’ responses to the surprise question. Third, the percentage of missing values for CRP is relatively high (42.1%), but additional sensitivity analyses revealed no differences between imputed and complete-case analyses. Fourth, due to the relatively high mortality rate in the selected patients, the nomogram might overestimate the risk of death in patients with advanced cancer types that have better overall survival (eg, breast cancer). However, cancer type prognosis was included as a variable in the model to neutralize this possible risk. Fifth, 54.9% of patients were enrolled at 1 hospital, which is the only participating university hospital (ie, tertiary hospital). In addition, although the internal-external validation of our model supports its external validity, it will be important to test the generalizability of our model by performing independent external validation using another data set. Sixth, our model may require regular updates due to developments in treatment options (eg, targeted therapy) or survival shifts in cancer care.

## Conclusions

This prognostic study found that a prediction model and nomogram including the surprise question, clinical characteristics (age, cancer type prognosis, visceral metastases, brain metastases, ECOG performance status, weight loss, pain, and dyspnea), and laboratory values (hemoglobin, CRP, and serum albumin) can support clinicians in more accurately identifying patients who are at risk of dying within 1 year. Further research on the nomogram should focus on external validation, feasibility, and its use for the initiation of advance care planning discussions with patients and relatives, which may aid in decision-making about desired care and medical treatment in the last period of patients’ lives.

## Notes

Supplement 1. **eBox 1.** Study Hospitals **eBox 2.** Candidate Predictors of Mortality **eBox 3.** Patient Example **eTable 1.** Characteristics of Patients in Study Hospitals **eTable 2.** C Statistic in Study Hospitals **eTable 3.** C Statistics of the Models and Additional Analyses **eFigure 1.** Calibration of Clinical Model per Study Hospital **eFigure 2.** Calibration of Extended Model per Study Hospital **eFigure 3.** Sensitivity Analyses **eFigure 4.** Nomogram of the Simple Model **eFigure 5.** Nomogram of the Clinical Model
eReferences

Supplement 2.
Data Sharing Statement

## References

[1] Ahmedzai SH, Costa A, Blengini C, ; International Working Group Convened by the European School of Oncology. A new international framework for palliative care. Eur J Cancer. 2004;40(15):2192-2200. doi:10.1016/j.ejca.2004.06.009 15454244

[2] Jordan K, Aapro M, Kaasa S, . European Society for Medical Oncology (ESMO) position paper on supportive and palliative care. Ann Oncol. 2018;29(1):36-43. doi:10.1093/annonc/mdx757 29253069

[3] Rietjens JAC, Sudore RL, Connolly M, ; European Association for Palliative Care. Definition and recommendations for advance care planning: an international consensus supported by the European Association for Palliative Care. Lancet Oncol. 2017;18(9):e543-e551. doi:10.1016/S1470-2045(17)30582-X 28884703

[4] Rome RB, Luminais HH, Bourgeois DA, Blais CM. The role of palliative care at the end of life. Ochsner J. 2011;11(4):348-352. 22190887 PMC3241069

[5] Glare PA, Sinclair CT. Palliative medicine review: prognostication. J Palliat Med. 2008;11(1):84-103. doi:10.1089/jpm.2008.9992 18370898

[6] Downar J, Goldman R, Pinto R, Englesakis M, Adhikari NKJ. The “surprise question” for predicting death in seriously ill patients: a systematic review and meta-analysis. CMAJ. 2017;189(13):E484-E493. doi:10.1503/cmaj.160775 28385893 PMC5378508

[7] Moss AH, Lunney JR, Culp S, . Prognostic significance of the “surprise” question in cancer patients. J Palliat Med. 2010;13(7):837-840. doi:10.1089/jpm.2010.0018 20636154

[8] Mudge AM, Douglas C, Sansome X, . Risk of 12-month mortality among hospital inpatients using the surprise question and SPICT criteria: a prospective study. BMJ Support Palliat Care. 2018;8(2):213-220. doi:10.1136/bmjspcare-2017-001441 29500239

[9] Supportive and Palliative Care Indicators Tool (SPICT). University of Edinburgh; 2019. Updated 2022. Accessed July 17, 2022. https://www.spict.org.uk/the-spict/

[10] Hui D. Prognostication of survival in patients with advanced cancer: predicting the unpredictable? Cancer Control. 2015;22(4):489-497. doi:10.1177/107327481502200415 26678976 PMC4769860

[11] Owusuaa C, Dijkland SA, Nieboer D, van der Heide A, van der Rijt CCD. Predictors of mortality in patients with advanced cancer—a systematic review and meta-analysis. Cancers (Basel). 2022;14(2):328. doi:10.3390/cancers14020328 35053493 PMC8774229

[12] Hui D, Park M, Liu D, . Clinician prediction of survival versus the Palliative Prognostic Score: which approach is more accurate? Eur J Cancer. 2016;64:89-95. doi:10.1016/j.ejca.2016.05.009 27372208 PMC4969216

[13] Collins GS, Reitsma JB, Altman DG, Moons KGM. Transparent Reporting of a Multivariable Prediction Model for Individual Prognosis or Diagnosis (TRIPOD): the TRIPOD statement. BMJ. 2015;350:g7594. doi:10.1136/bmj.g7594 25569120

[14] Oken MM, Creech RH, Tormey DC, . Toxicity and response criteria of the Eastern Cooperative Oncology Group. Am J Clin Oncol. 1982;5(6):649-655. doi:10.1097/00000421-198212000-00014 7165009

[15] Williamson A, Hoggart B. Pain: a review of three commonly used pain rating scales. J Clin Nurs. 2005;14(7):798-804. doi:10.1111/j.1365-2702.2005.01121.x 16000093

[16] Neo HY, Xu HY, Wu HY, Hum A. Prediction of poor short-term prognosis and unmet needs in advanced chronic obstructive pulmonary disease: use of the two-minute walking distance extracted from a six-minute walk test. J Palliat Med. 2017;20(8):821-828. doi:10.1089/jpm.2016.0449 28353374

[17] Charlson ME, Pompei P, Ales KL, MacKenzie CR. A new method of classifying prognostic comorbidity in longitudinal studies: development and validation. J Chronic Dis. 1987;40(5):373-383. doi:10.1016/0021-9681(87)90171-8 3558716

[18] Katagiri H, Takahashi M, Wakai K, Sugiura H, Kataoka T, Nakanishi K. Prognostic factors and a scoring system for patients with skeletal metastasis. J Bone Joint Surg Br. 2005;87(5):698-703. doi:10.1302/0301-620X.87B5.15185 15855375

[19] Tomita K, Kawahara N, Kobayashi T, Yoshida A, Murakami H, Akamaru T. Surgical strategy for spinal metastases. Spine (Phila Pa 1976). 2001;26(3):298-306. doi:10.1097/00007632-200102010-00016 11224867

[20] Peduzzi P, Concato J, Kemper E, Holford TR, Feinstein AR. A simulation study of the number of events per variable in logistic regression analysis. J Clin Epidemiol. 1996;49(12):1373-1379. doi:10.1016/S0895-4356(96)00236-3 8970487

[21] van Buuren S. *Flexible Imputation of Missing Data*. 1st ed. Chapman and Hall/CRC; 2012.

[22] Steyerberg EW, Harrell FE Jr. Prediction models need appropriate internal, internal-external, and external validation. J Clin Epidemiol. 2016;69:245-247. doi:10.1016/j.jclinepi.2015.04.005 25981519 PMC5578404

[23] Nieboer D. Prediction model for patients with advance disease in oncology. Shinyapps. October 25, 2019. Accessed October 27, 2022. https://dnieboer.shinyapps.io/nomogram

[24] Proctor MJ, Morrison DS, Talwar D, . A comparison of inflammation-based prognostic scores in patients with cancer. a Glasgow Inflammation Outcome study. Eur J Cancer. 2011;47(17):2633-2641. doi:10.1016/j.ejca.2011.03.028 21724383

[25] Manz CR, Chen J, Liu M, . Validation of a machine learning algorithm to predict 180-day mortality for outpatients with cancer. JAMA Oncol. 2020;6(11):1723-1730. doi:10.1001/jamaoncol.2020.4331 32970131 PMC7516810

[26] White N, Kupeli N, Vickerstaff V, Stone P. How accurate is the ‘surprise question’ at identifying patients at the end of life? a systematic review and meta-analysis. BMC Med. 2017;15(1):139. doi:10.1186/s12916-017-0907-4 28764757 PMC5540432

[27] Lefkowits C, Chandler C, Sukumvanich P, . Validation of the ‘surprise question’ in gynecologic oncology: comparing physicians, advanced practice providers, and nurses. Gynecol Oncol. 2016;141 (suppl 1):128. doi:10.1016/j.ygyno.2016.04.339 26867989

