# Supplementary material for: Development of a Clinical Prediction Model for 1-Year Mortality in Patients With Advanced Cancer
Source: JAMA Netw Open. 2022 Nov 30;5(11):e2244350. doi: 10.1001/jamanetworkopen.2022.44350 (PMC9713606; doi:10.1001/jamanetworkopen.2022.44350)
Supplement: Supplement 1. — eBox 1. Study Hospitals eBox 2. Candidate Predictors of Mortality eBox 3. Patient Example eTable 1. Characteristics of Patients in Study Hospitals eTable 2. C Statistic in Study Hospitals eTable 3. C Statistics of the Models and Additional Analyses eFigure 1. Calibration of Clinical Model per Study Hospital eFigure 2. Calibration of Extended Model per Study Hospital eFigure 3. Sensitivity Analyses eFigure 4. Nomogram of the Simple Model eFigure 5. Nomogram of the Clinical Model eReferences [file jamanetwopen-e2244350-s001.pdf]

Supplemental Online Content

Owusuaa C, van der Padt-Pruijsten A, Drooger JC, et al. Development of a clinical prediction model for 1-year mortality in patients with advanced cancer. *JAMA Netw Open*. 2022;5(11):e2244350. doi:10.1001/jamanetworkopen.2022.44350

eBox 1. Study Hospitals

eBox 2. Candidate Predictors of Mortality

eBox 3. Patient Example

eTable 1. Characteristics of Patients in Study Hospitals

eTable 2. C Statistic in Study Hospitals

eTable 3. C Statistics of the Models and Additional Analyses

eFigure 1. Calibration of Clinical Model per Study Hospital

eFigure 2. Calibration of Extended Model per Study Hospital

eFigure 3. Sensitivity Analyses

eFigure 4. Nomogram of the Simple Model

eFigure 5. Nomogram of the Clinical Model

eReferences

This supplemental material has been provided by the authors to give readers additional information about their work.

© 2022 Owusuaa C et al. *JAMA Network Open*.

eBox 1. Study Hospitals

1. Erasmus MC	University hospital
2. Maasstad Hospital Rotterdam	Teaching hospital
3. Ikazia Hospital Rotterdam	Teaching hospital
4. Amphia	Teaching hospital
5. Van Weel Bethesda Hospital	Local hospital
6. Admiraal de Ruyter Hospital	Local hospital

© 2022 Owusuaa C et al. *JAMA Network Open*.

eBox 2. Candidate Predictors of Mortality

Predictors	References
Surprise question	[1]
Clinical characteristics	
Age	[1]
Sex	[1]
Comorbidity	[1]
Cancer type	[1-3]
Performance status	[1]
Visceral metastases	[1]
Brain metastases	[1]
Food intake	[1]
Pain	[1]
Weight loss	[1]
Fatigue	[1]
Dyspnea	[1]
	[1]
Laboratory values	
Hemoglobin	[1]
C-reactive protein	[1]
Serum albumin	[1]

© 2022 Owusuaa C et al. *JAMA Network Open*.

eBox 3. Patient Example

**Male patient**<br>Surprise question: No<br>Age: 63 years<br>Cancer type prognosis: Intermediate or poor (Lung cancer)<br>WHO performance status: grade 2<br>Weight loss in the past 6 months: No<br>Pain score on a scale of 0-10: 2<br>Dyspnea: CTCAE grade 2<br>Brain metastases: No<br>Visceral metastases: Yes (liver metastases)<br>Hemoglobin 5.9 mmol/L<br>C-reactive protein 10 mg/L<br>Serum albumin: 28 g/L<br><br>**1-year mortality risk according to:**<br>Simple model: 64%<br>Clinical model: 89%<br>Extended model: 97%

© 2022 Owusuaa C et al. *JAMA Network Open*.

eTable 1. Characteristics of Patients in Study Hospitals

	Erasmus MC<br>No (%)	Ikazia Hospital Rotterdam<br>No (%)	Maasstad Hospital Rotterdam<br>No (%)	Amphia<br>No (%)	Van Weel Bethesda Hospital<br>No (%)	Admiraal de Ruyter Hospital<br>No (%)
No. of patients	476 (100)	133 (100)	77 (100)	104 (100)	29 (100)	48 (100)
Age, median (IQR), y	63 [54–70]	70 [61–74]	69 [58–75]	66 [58–72]	67 [63–72]	72.5 [67–77]
Sex, male	233 (48.9)	59 (44.4)	36 (46.8)	47 (45.2)	16 (55.2)	20 (41.7)
Clinician response of *no* to surprise question[a]	254 (53.9)	64 (49.6)	32 (42.7)	61 (59.2)	19 (65.5)	15 (31.9)
Respondents to surprise question						
Medical specialists	391 (82.1)	126 (94.7)	71 (92.2)	102 (98.1)	29 (100.0)	48 (100.0)
Nurse practitioners	54 (11.3)	0 (0.0)	0 (0.0)	1 (1.0)	0 (0.0)	0 (0.0)
Residents	31 (6.5)	7 (5.3)	6 (7.8)	1 (1.0)	0 (0.0)	0 (0.0)
ECOG performance status						
0	143 (30.0)	49 (36.8)	16 (21.1)	29 (27.9)	9 (31.0)	18 (39.1)
1	263 (55.3)	47 (35.3)	40 (52.6)	53 (51.0)	9 (31.0)	20 (43.5)
2+	70 (14.7)	37 (27.8)	20 (26.0)	22 (21.2)	11 (37.9)	8 (16.7)
Cancer type						
Breast	91 (19.1)	42 (31.6)	23 (29.9)	15 (14.4)	4 (13.8)	16 (33.3)
Lung	74 (15.5)	25 (18.8)	11 (14.3)	54 (51.9)	2 (6.9)	7 (14.6)
Gastro-intestinal	54 (11.3)	34 (25.6)	16 (20.8)	11 (10.6)	10 (34.5)	7 (14.6)
Prostate	21 (4.4)	18 (13.5)	15 (19.5)	9 (8.7)	6 (20.7)	7 (14.6)
Melanoma	57 (12.0)	0 (0.0)	1 (1.3)	2 (1.9)	0 (0.0)	0 (0.0)
Gynecological	33 (6.9)	4 (3.0)	3 (3.9)	7 (6.7)	2 (6.9)	4 (8.3)
Pancreas	23 (4.8)	4 (3.0)	6 (7.8)	3 (2.9)	2 (6.9)	2 (4.2)
Thyroid	5 (1.1)	0 (0.0)	0 (0.0)	0 (0.0)	0 (0.0)	0 (0.0)
All other types	118 (24.8)	6 (4.5)	2 (2.6)	3 (2.9)	3 (10.3)	5 (10.4)
Cancer type prognosis						
Good	117 (24.6)	60 (45.1)	38 (49.4)	24 (23.1)	10 (34.5)	23 (47.9)
Intermediate or poor	359 (75.4)	73 (54.9)	39 (50.6)	80 (76.9)	19 (65.5)	25 (52.1)
Visceral metastases	204 (42.9)	46 (34.6)	34 (44.2)	33 (31.7)	13 (44.8)	17 (35.4)
Brain metastases	34 (7.1)	10 (7.5)	5 (6.5)	17 (16.3)	0 (0.0)	0 (0.0)
Subcutaneous or cutaneous metastases	32 (6.7)	3 (2.3)	4 (5.2)	1 (1.0)	0 (0.0)	0 (0.0)
Food intake						
Normal	362 (76.9)	102 (77.9)	51 (69.9)	77 (74.8)	17 (58.6)	31 (66.0)
Lightly reduced	83 (17.6)	22 (16.8)	14 (19.2)	20 (19.4)	9 (31.0)	11 (23.4)
Strongly reduced	26 (5.5)	7 (5.3)	8 (11.0)	6 (5.8)	3 (10.3)	5 (10.6)
Weight loss, median (IQR), kg	0 [0–2]	0 [0–2.5]	0 [0–2.5]	0 [0–2]	0 [0–5]	0 [0–3.3]
Pain score, median (IQR)[b]	0 [0–3]	0 [0–3]	0 [0–5]	0 [0–1]	3 [0–5]	0 [0–2]
Dyspnea level[c]						
Grade 0	338 (71.0)	83 (62.4)	41 (53.2)	64 (61.5)	14 (48.3)	30 (62.5)
Grade 1	111 (23.3)	35 (26.3)	21 (27.3)	32 (30.8)	10 (34.5)	16 (33.3)
Grade 2+	27 (5.7)	15 (11.3)	15 (19.5)	8 (7.7)	5 (17.2)	2 (4.2)
Fatigue level[d]						
Grade 0	149 (31.3)	40 (30.1)	18 (23.4)	26 (25.2)	6 (20.7)	17 (35.4)
Grade 1	257 (54.0)	68 (51.1)	49 (63.6)	65 (63.1)	18 (62.1)	27 (56.2)
Grade 2+	70 (14.7)	25 (18.8)	10 (13.0)	12 (11.5)	5 (17.2)	4 (8.3)
Charlson \comorbidity Index score						
0	336 (70.6)	72 (54.1)	49 (63.6)	63 (60.6)	18 (62.1)	32 (66.7)
1	98 (20.6)	49 (36.8)	16 (20.8)	27 (26.0)	7 (24.1)	11 (22.9)
2+	42 (8.8)	12 (9.0)	12 (15.6)	14 (13.3)	4 (13.8)	5 (10.4)
Hemoglobin, median (IQR), mmol/L	7.7 [6.9–8.7]	8.0 [7.1–8.5]	7.7 [7.0–8.4]	7.8 [7.1– 8.4]	8.3 [7.0–8.6]	7.6 [7.0–8.1]

© 2022 Owusuaa C et al. *JAMA Network Open*.

C-reactive protein, median (IQR), mg/L	5.0 [1.9–18.3]	20.0 [6.5–60.3]	11.0 [3.0–45.0]	7.0 [3.0–22.0]	12.7 [2.9–40.8]	7.6 [5.6–39.0]
Serum albumin, median (IQR), g/L	42 [39–44]	42 [39–43]	33 [29–36]	41 [38–43]	41 [37–43]	35 [33–38]
Dead at 1 y	191 (40.1)	64 (48.1)	33 (42.9)	47 (45.2)	11 (37.9)	15 (31.2)

Abbreviations: ECOG, Eastern Cooperative Oncology Group.

[a]The surprise question was, "Would I be surprised if this patient died in the next year?"

[b]Pain was assessed using an 11-point numerical rating scale (score range, 0-10, with 0 indicating no pain and 10 indicating the worst pain possible).

[c,d]Dyspnea and fatigue levels were assessed using the Common Terminology Criteria for Adverse Events, version 4.0. For dyspnea, the range was 0 to 4, with 0 indicating no dyspnea and 4 indicating life-threatening dyspnea; for fatigue, the range was 0 to 3, with 0 indicating no fatigue and 3 indicating fatigue that limits self-care activities of daily living.

© 2022 Owusuaa C et al. *JAMA Network Open*.

eTable 2. C Statistic in Study Hospitals

**Hospital**	**Clinical model** c-statistic (95% CI)	**Extended model** c-statistic (95% CI)
Erasmus MC	0.74 (0.70–0.78)	0.76 (0.73–0.79)
Ikazia Hospital Rotterdam	0.79 (0.74–0.84)	0.80 (0.75–0.85)
Maasstad Hospital Rotterdam	0.79 (0.71–0.86)	0.80 (0.72–0.87)
Amphia	0.65 (0.56–0.73)	0.67 (0.59–0.75)
Van Weel Bethesda Hospital	0.74 (0.60–0.88)	0.83 (0.71–0.95)
Admiraal de Ruyter Hospital	0.75 (0.61–0.88)	0.75 (0.62–0.88)

CI: confidence interval

© 2022 Owusuaa C et al. *JAMA Network Open*.

eTable 3. C Statistics of the Models and Additional Analyses

**Model / analyses**	**C-statistic (95% CI)**
Simple model	0.69 (0.67–0.71)
Clinical model	0.76 (0.73–0.78)
Extended model	0.78 (0.76–0.80)
Only clinical characteristics	0.70 (0.68–0.73)
Only laboratory values	0.71 (0.68–0.74)
Surprise question + laboratory values	0.77 (0.74–0.79)

CI: confidence interval.

© 2022 Owusuaa C et al. *JAMA Network Open*.

eFigure 1. Calibration of Clinical Model per Study Hospital

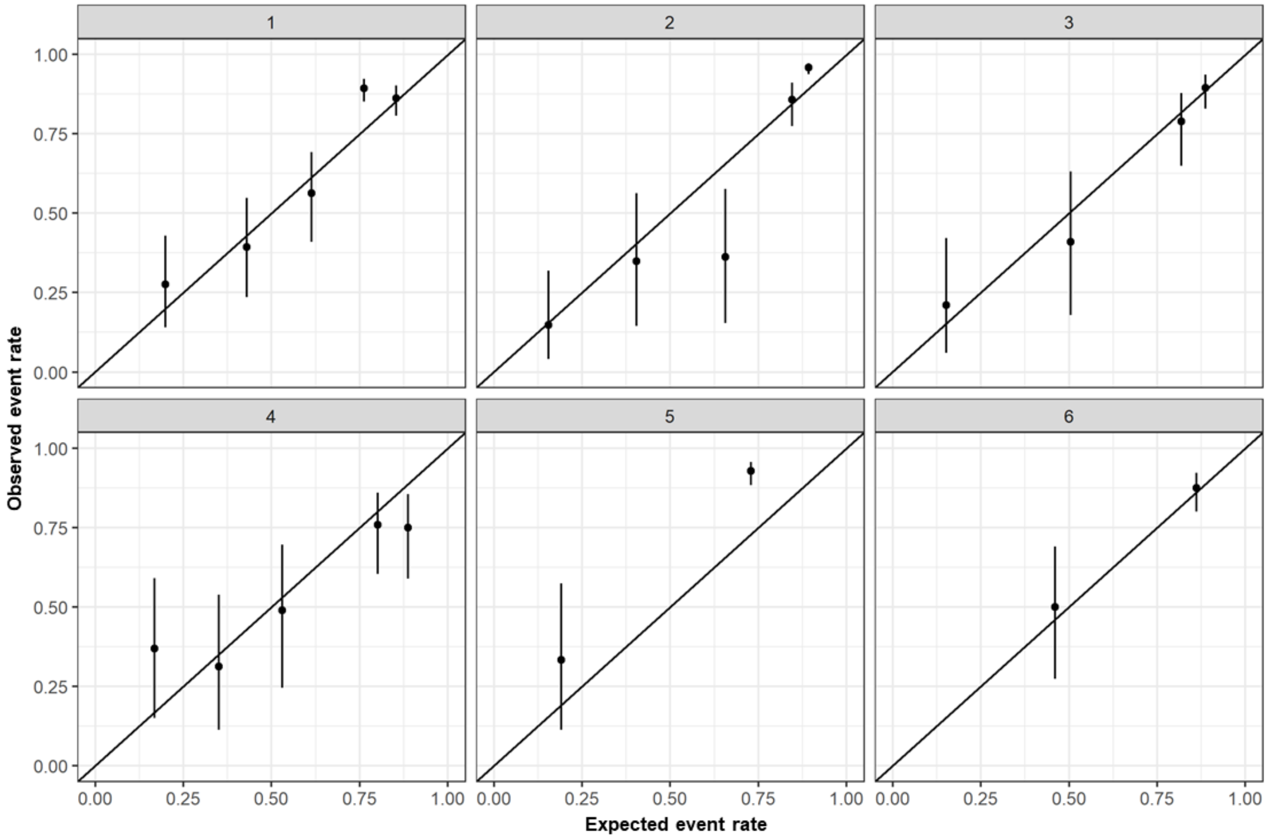

1: Erasmus MC; 2: Ikazia Hospital Rotterdam; 3: Maasstad Hospital Rotterdam; 4: Amphia; 5: Van Weel Bethesda Hospital; 6: Admiraal de Ruyter Hospital

© 2022 Owusuaa C et al. *JAMA Network Open*.

eFigure 2. Calibration of Extended Model per Study Hospital

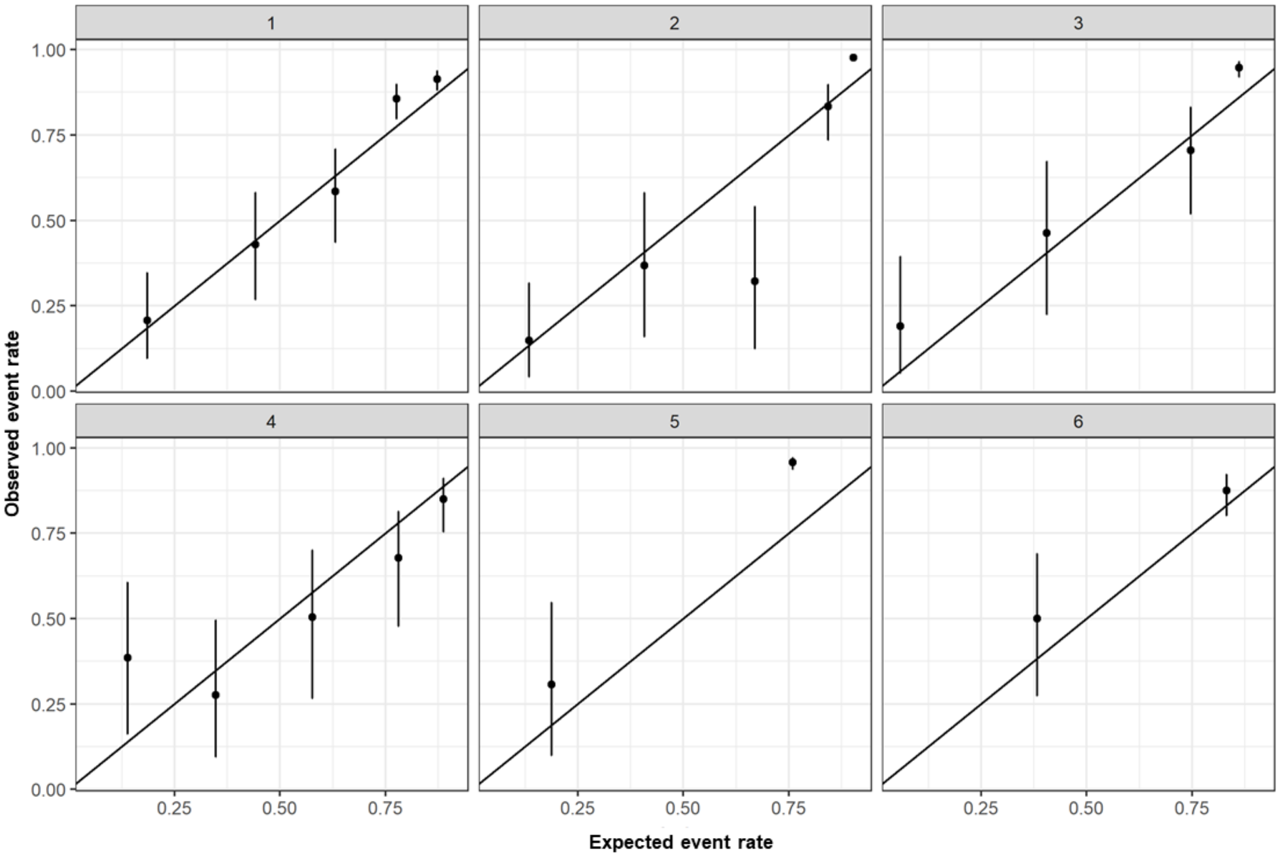

1: Erasmus MC; 2: Ikazia Hospital Rotterdam; 3: Maasstad Hospital Rotterdam; 4: Amphia; 5: Van Weel Bethesda Hospital; 6: Admiraal de Ruyter Hospital

© 2022 Owusuaa C et al. *JAMA Network Open*.

eFigure 3. Sensitivity Analyses

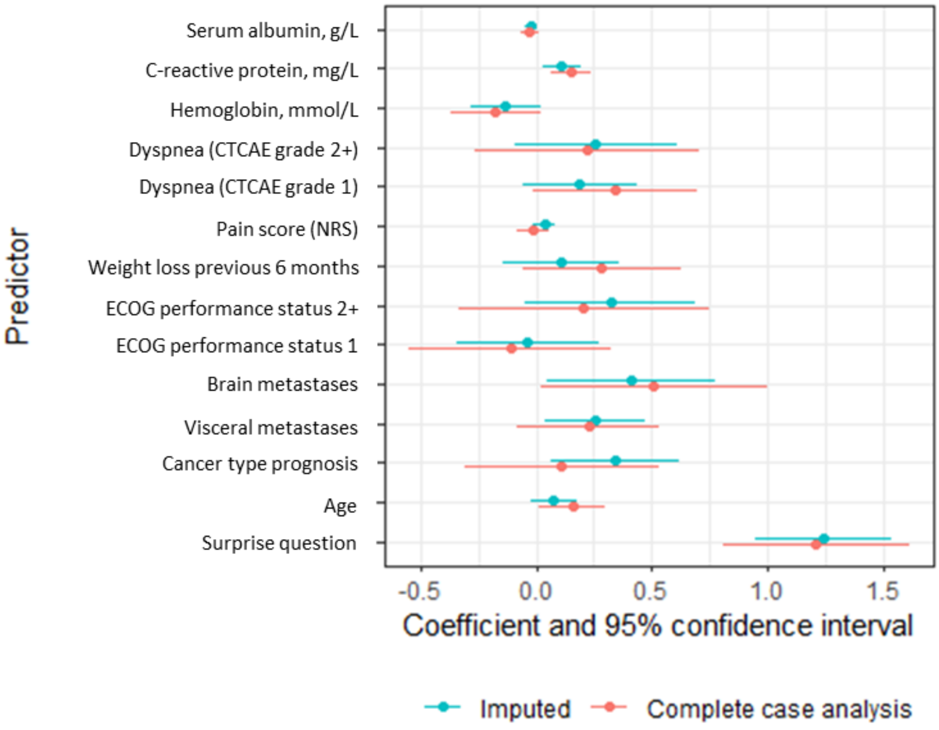

© 2022 Owusuaa C et al. *JAMA Network Open*.

eFigure 4. Nomogram of the Simple Model

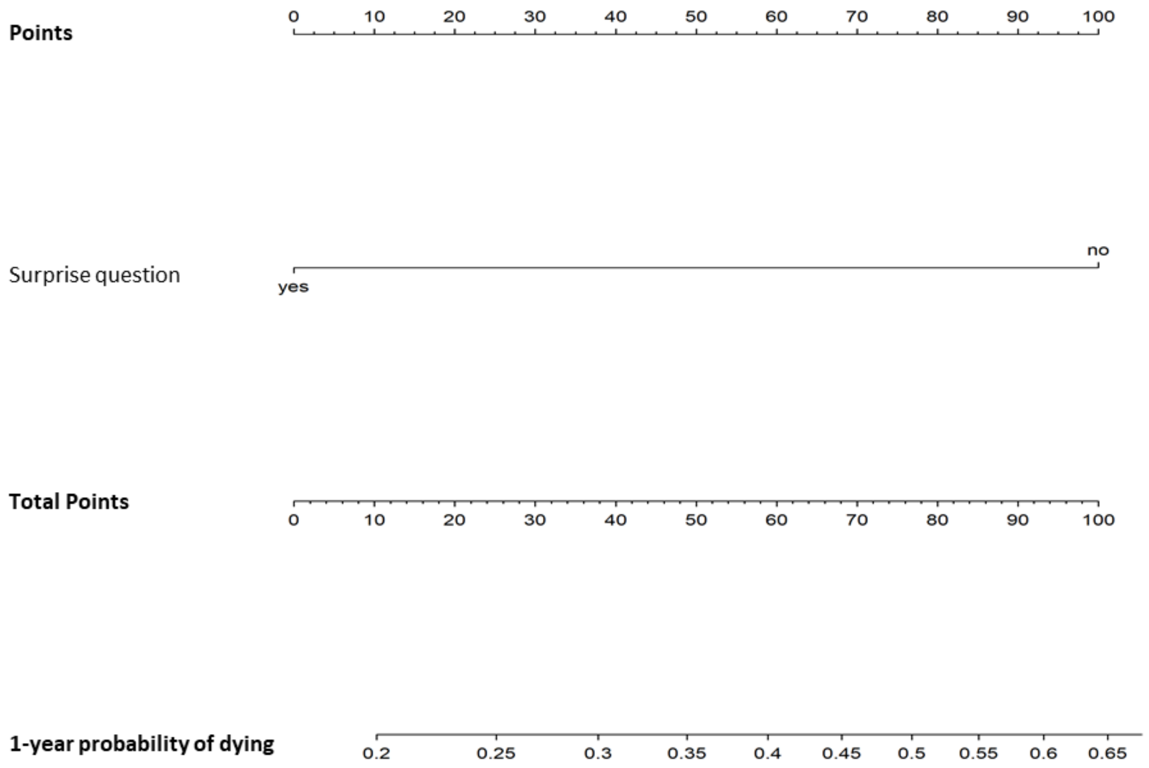

The surprise question was: "Would I be surprised if this patient died within the next year?" Instructions for use of the nomogram: (1) locate the answer to the surprise question, (2) draw a straight downwards to the total points axis, and (3) draw a line straight down to the 1-year mortality probability axis to find the patient's risk of dying within 1 year.

© 2022 Owusuaa C et al. *JAMA Network Open*.

eFigure 5. Nomogram of the Clinical Model

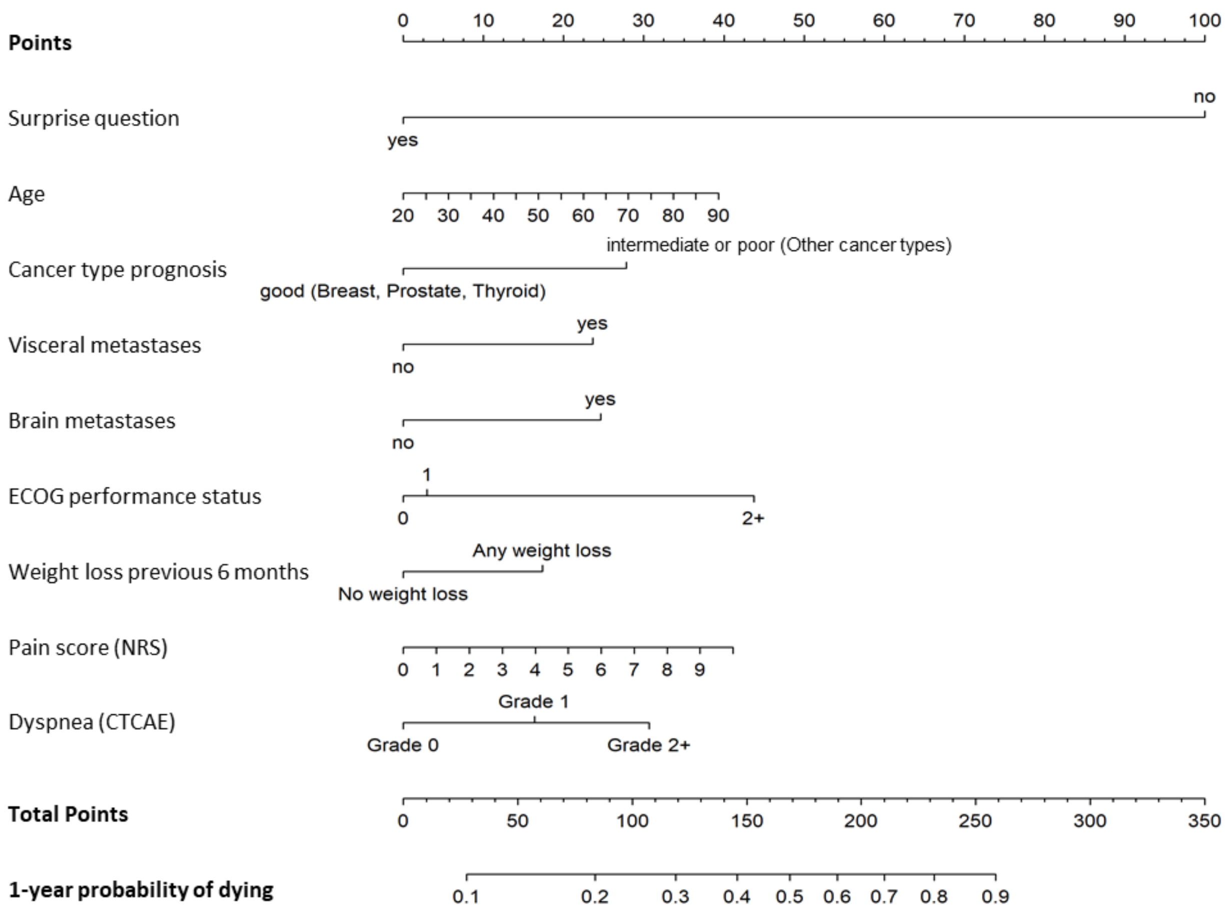

Abbreviations: ECOG, Eastern Cooperative Oncology Group; NRS, Numerical Rating Scale, CTCAE: Common Terminology Criteria for Adverse Events.
The surprise question was: "Would I be surprised if this patient died within the next year?" Instructions for use of the nomogram: (1) locate the answer to the surprise question, (2) draw a straight upwards to the points axis, (3) repeat this procedure the other 8 predictors (age, cancer type prognosis, visceral metastases, brain metastases, ECOG performance status, weight loss, pain score, and dyspnea), (4) sum the points for all the predictors on the total points axis, and (5) draw a line straight down to the 1-year mortality probability axis to find the patient's risk of dying within 1 year.

© 2022 Owusuaa C et al. *JAMA Network Open*.

eReferences

1. Owusuaa C, Dijkland SA, Nieboer D, van der Heide A, van der Rijt CCD. Predictors of Mortality in Patients with Advanced Cancer-A Systematic Review and Meta-Analysis. Cancers (Basel). 2022;14(2).
2. Katagiri H, Takahashi M, Wakai K, Sugiura H, Kataoka T, Nakanishi K. Prognostic factors and a scoring system for patients with skeletal metastasis. J Bone Joint Surg Br. 2005;87(5):698-703.
3. Tomita K, Kawahara N, Kobayashi T, Yoshida A, Murakami H, Akamaru T. Surgical strategy for spinal metastases. Spine (Phila Pa 1976). 2001;26(3):298-306.

© 2022 Owusuaa C et al. *JAMA Network Open*.
