# Supplementary material for: Development of a Clinical Prediction Model for 1-Year Mortality in Patients With Advanced Cancer
Source: JAMA Netw Open. 2022 Nov 30;5(11):e2244350. doi: 10.1001/jamanetworkopen.2022.44350 (PMC9713606; doi:10.1001/jamanetworkopen.2022.44350)
Supplement: Supplement 2. — Data Sharing Statement [file jamanetwopen-e2244350-s002.pdf]

Data Sharing Statement

Owusuaa. Development of a Clinical Prediction Model for 1-Year Mortality in Patients With Advanced Cancer. *JAMA Netw Open*. Published November 30, 2022.
doi:10.1001/jamanetworkopen.2022.44350

Data
Data available: Yes
Data types: Participant data with identifiers
How to access data: https://doi.org/10.17026/dans-zu5-gthp
When available: With publication

Supporting Documents
Document types: None

Additional Information
Who can access the data: Anyone requesting the data
Types of analyses: For a specified purpose.
Mechanisms of data availability: After approval of a proposal
